# Association of Postoperative Atrial Fibrillation Duration after Coronary Artery Bypass Grafting with Poor Postoperative Outcomes

**DOI:** 10.31083/j.rcm2503098

**Published:** 2024-03-08

**Authors:** Haokai Qin, Enzehua Xie, Zhan Peng, Xiubin Yang, Kun Hua

**Affiliations:** ^1^Department of Cardiovascular Surgery, Beijing Anzhen Hospital, Capital Medical University, 100029 Beijing, China; ^2^Department of Cardiovascular Surgery, Fuwai Hospital, State Key Laboratory of Cardiovascular Disease, Chinese Academy of Medical Sciences & Peking Union Medical College/National Center for Cardiovascular Diseases, 100037 Beijing, China

**Keywords:** postoperative atrial fibrillation, coronary artery bypass graft surgery, postoperative outcomes

## Abstract

**Background:**

Postoperative atrial fibrillation (POAF) has long been associated with poor perioperative outcomes after coronary artery bypass grafting (CABG). In this study, we aimed to investigate the effect of prolonged POAF durations on perioperative outcomes of CABG.

**Methods:**

This retrospective cohort study examined CABG patients enrolled at Beijing Anzhen Hospital from January 2018 to September 2021. We compared patients with POAF durations ≥48 hours to patients with POAF durations <48 hours. Primary outcomes were in-hospital mortality, stroke, acute respiratory failure (ARF), acute kidney injury (AKI), and significant gastrointestinal bleeding (GIB); secondary outcomes were postoperative length of stay (LOS) and intensive care unit (ICU) duration. Associations between primary outcomes and POAF duration were determined using logistic regression and restricted cubic spline analyses. Differences in baseline characteristics were controlled using propensity score matching (PSM) and inverse probability of treatment weighting (IPTW).

**Results:**

Out of 11,848 CABG patients, 3604 (30.4%) had POAF, while 1131 (31.4%) had it for a duration of ≥48 hours. ARF (adjusted odds ratio [OR]: 2.96, 95% confidence interval [CI]: 1.47–6.09), AKI (adjusted OR: 2.37, 95% CI: 1.42–3.99), and significant GIB (adjusted OR: 2.60, 95% CI: 1.38–5.03) were associated with POAF durations ≥48 hours; however, neither in-hospital mortality (adjusted OR: 1.60, 95% CI: 0.97–2.65) nor stroke (adjusted OR: 1.28, 95% CI: 0.71–2.34) was. These results remained even following PSM and IPTW analyses.

**Conclusions:**

POAF durations longer than 48 hours were independently associated with poorer perioperative recovery from CABG, with respect to the occurrence of ARF, AKI, and GIB, as well as a longer postoperative LOS and ICU duration. However, it was not associated with greater in-hospital mortality or stroke occurrence. All these findings suggest that postoperative monitoring of POAF and positive intervention after detection may be more helpful in optimizing post-CABG patient outcomes.

## 1. Introduction

The onset of postoperative atrial fibrillation (POAF) is one of the most common complications after cardiac surgery, occurring in approximately 30% of patients who undergo coronary artery bypass grafting (CABG) [1, 2]. Although most POAF is resolved within 24 hours [3], it is still related to the occurrence of many adverse perioperative events [4, 5]. Previous studies have shown that in approximately 25% of POAF patients, sinus rhythm (SR) cannot be restored after discharge [6]. Furthermore, prolonged duration of POAF after cardiac surgery was independently associated with high long-term mortality [7], which indicates that different POAF durations could lead to differing outcomes in CABG patients. However, few studies have been conducted to fully elucidate the precise relationship between POAF duration and perioperative outcomes after CABG.

Thus, to our knowledge, this study is the first to explore, in a large cohort, the effects of prolonged POAF duration on in-hospital mortality, stroke, and other important perioperative outcomes among CABG patients.

## 2. Methods

### 2.1 Establishing the Study Populations 

**Fig. 1.**
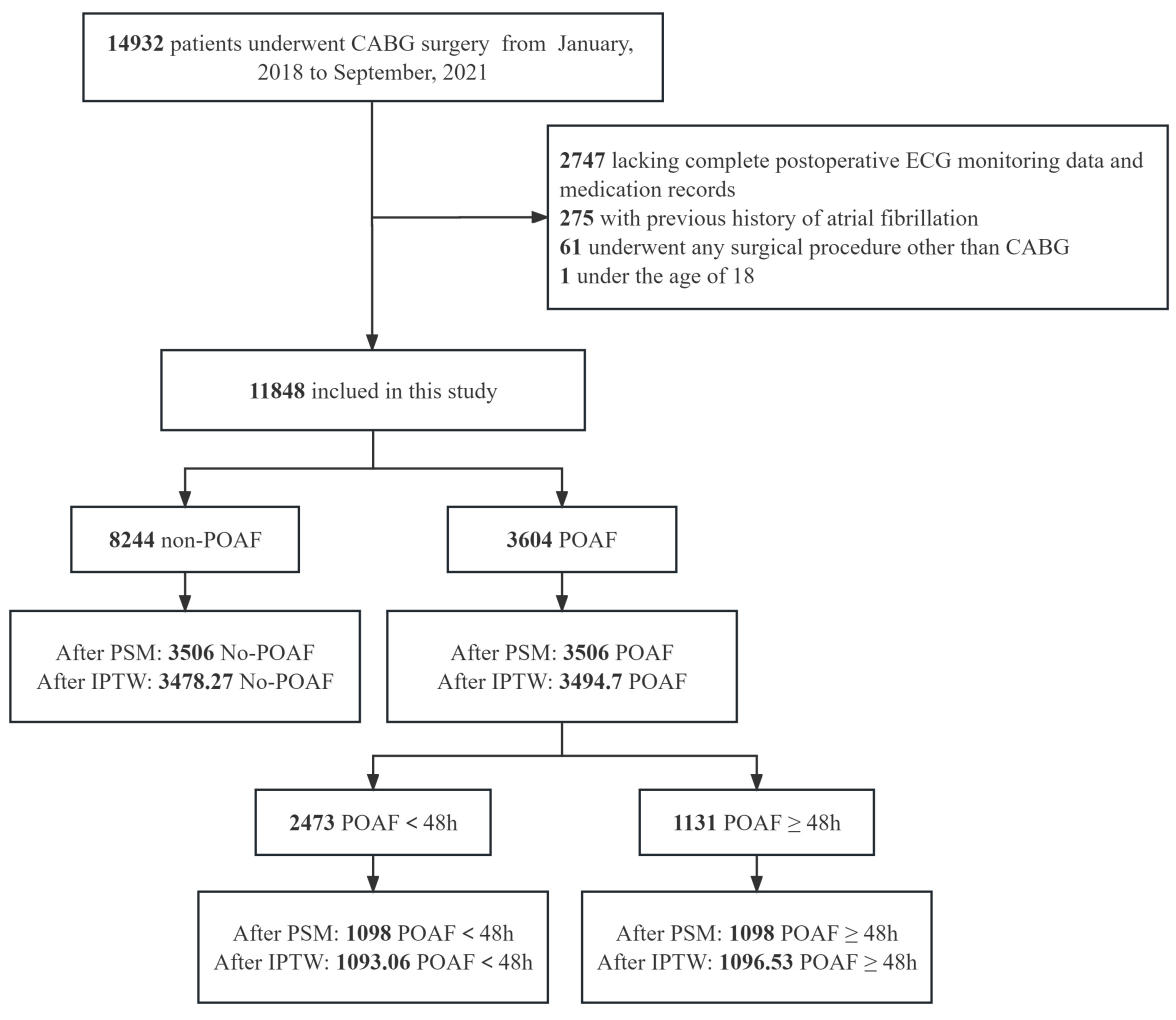
**Study flowchart**. CABG, coronary artery bypass grafting; ECG, electrocardiography; POAF, postoperative atrial fibrillation; PSM, propensity score matching; IPTW, inverse probability of treatment weighting.

In this retrospective study, we consecutively collected a total of 14,932 patients, who underwent isolated CABG at Beijing Anzhen Hospital between January 2018 and September 2021. The exclusion criteria were as follows: History of atrial fibrillation (AF), non-CABG surgical procedures, missing postoperative continuous electrocardiography (ECG) and medication records, or being <18 years old. This resulted in 3084 patients being excluded from this study, leaving a total of 11,848 patients. Fig. 1 depicts the flowchart of the included and excluded patients.

POAF was diagnosed using a continuous ECG monitor, whereby it was defined after surgery lasting at least 5 minutes as either newly onset atrial fibrillation or atrial flutter, and treatment was required in accordance with the definition provided by the Society of Thoracic Surgeons (STS) National Database [8]. Patients with POAF were treated with amiodarone, and electrical cardioversion was administered to patients with POAF who developed hemodynamic instability. All patients were first transferred to the ICU postsurgery, where POAF occurrence was continuously monitored during their stay duration, as well as during the first 4 days after being transferred back to the general ward. If POAF was detected, patients were continually monitored until a normal SR returned or they were discharged. The POAF duration was defined as the interval between the first day and the last day that POAF occurred (continuous or intermittent POAF during the monitoring time) [7]. Ultimately, patients were first divided into 2 groups: non-POAF (n = 8244) and POAF (n = 3604). Then, the POAF group was further divided into 2 subgroups based on whether POAF had occurred for longer than 48 hours: POAF durations shorter than 48 hours (n = 2473) and longer than 48 hours (n = 1131). The 48-hour cutoff was based on AF, with that duration considered the nodal point for initiating long-term anticoagulation therapies [9].

### 2.2 Surgical Techniques for Isolated CABG

After anesthesia, the patients who underwent CABG had access through a sternotomy. Off-pump patients were fully heparinized at the completion of harvesting the conduits, while the heart was stabilized when conducting the grafts using the Octopus tissue stabilizer system (cardiopulmonary bypass was established first in on-pump patients, and the Octopus tissue stabilizer system was not used). After all grafts were performed, proximal anastomosis was performed on the aorta using partial clamping. The heparinized state was reversed using protamine and the chest was closed (on-pump patients were firstly withdrawn from cardiopulmonary bypass, and after the heart resumed beating, the chest was closed).

### 2.3 Data Collection and Patient Outcomes

Patient data were collected using an electronic medical records system, including demographics, comorbidities, in-hospital outcomes, medications, preoperative laboratory, echocardiography, and continuous ECG monitoring data. The primary outcomes measured were in-hospital mortality, stroke, acute respiratory failure (ARF), acute kidney injury (AKI), and significant gastrointestinal bleeding (GIB). In-hospital mortality was defined as death from any cause during hospitalization. Stroke was defined as a permanent neurological deficit with imaging evidence of cerebral artery occlusion, as diagnosed by neurologists. ARF was defined as arterial partial $O_{2}$ pressure (PaO2) remaining ≤60 mmHg after oxygen therapy, as measured via nasal cannula, or PaO2/inhaled partial pressure of oxygen remaining ≤200 mmHg after oxygen therapy, as measured via venturi mask, with or without pH <7.35 and arterial partial ${\mathrm{CO}}_{2}$ pressure >45 mmHg [10]. AKI was defined as a 0.3 mg/dL or 50% increase in serum creatinine from baseline or urine volume <0.5 mL/kg/h for 6 hours [11]. Significant GBI was defined as nasogastric drainage, yielding obviously red or coffee-ground-appearing blood, as well as melena (black, tarry stools), hematemesis (blood vomiting), or hematochezia (passage of fresh blood through the anus), along with hemoglobin levels decreasing >2 g/mL/day, and transfusion of blood products being required [12]. The measured secondary outcomes were postoperative length of stay (LOS) and intensive care unit (ICU) duration. Considering that the inclusion of outcomes that partially occurred during POAF duration may blur the relationship between POAF duration and outcomes; thus, to clarify whether the differences in outcomes were caused by “prolonged duration of POAF”, outcomes that occurred during POAF duration in the short-duration group and within 48 hours of POAF duration in the long-duration group were excluded.

### 2.4 Statistical Analysis

The normality distribution test for continuous variables was performed using the Kolmogorov‒Smirnov test. The results are presented as the mean ± standard deviation for normally distributed continuous variables and were compared between two groups by Student’s *t* test. When continuous variables were not normally distributed, interquartile ranges (25th–75th percentiles) were used, and groups were compared using the Mann‒Whitney U test. Categorical data are presented as n (%) and were compared using chi-squared tests. Missing data are absent completely at random with a missing degree of less than 1%. Mean imputation was used to fill in the missing baseline data values.

Analyses were performed by propensity score matching (PSM) and inverse probability of treatment weighting (IPTW) to control the differences in baseline characteristics of patients between the non-POAF group and POAF group and between the group with POAF durations shorter than 48 hours and the group with POAF durations longer than 48 hours to account for selection bias and potential confounding factors in outcome comparisons between the groups. A propensity score for each patient was calculated using multivariable logistic regression. PSM was performed using the 1:1 nearest neighbor matching method and optimal matching with a caliper width of 0.25 standard deviations. Regarding IPTW, the inverse propensity score served as the weight for patients with POAF or POAF durations longer than 48 hours, and the inverse of 1 minus the propensity score served as the weight for patients without POAF or POAF durations shorter than 48 hours.

A restricted cubic spline (RCS) model with four knots (at the 5th, 35th, 65th, and 95th percentiles) and including major confounding variates associated with adverse perioperative outcomes after cardiac surgery, including age, male sex, body mass index (BMI), history of hypertension, diabetes, chronic kidney disease (CKD), stroke and cardiac surgery, operation time and preoperative level of left atrial diameter (LAD), left ventricular ejection fraction (LVEF), creatinine (Cr), creatine kinase MB (CK-MB), troponin I (TnI), and brain natriuretic peptide (BNP), was used to explore the nonlinear dose‒response relationship between POAF duration and each primary outcome. Univariable and multivariable logistic regression models were used to estimate the odds ratios (ORs) and 95% confidence intervals (CIs) of the independent association between POAF or prolonged POAF duration and the occurrence of primary outcomes. PSM, IPTW, and multivariable logistic regression analyses were performed by including the same confounding variables in the RCS. In addition, we used logistic regression analysis to analyze the risk factors that cause a prolonged POAF duration.

Statistical analyses were performed by R software (version 4.2.2, R Foundation for Statistical Computing, Vienna, Austria). The statistical significance level was set at two-tailed *p* < 0.05.

## 3. Results

### 3.1 Patient Baseline Characteristics Were All Balanced between Non-POAF and POAF, as well as between POAF Durations Shorter than 48 Hours and POAF Durations Longer than 48 Hours

Out of the 11,848 patients included in this study, 75.6% were male. POAF was detected in 3604 (30.4%) patients, which was consistent with previous studies regarding the POAF prevalence among large patient cohorts who had undergone CABG [1, 2, 13]; POAF durations longer than 48 hours were observed in 1131 (31.4%) patients.

With respect to the patient baseline parameters, significant differences were observed between the non-POAF group and POAF groups for age, male sex, the prevalence of hypertension, chronic obstructive pulmonary disease (COPD), stroke, hyperlipidemia, prior cardiac surgery, the rate of on-pump CABG, postoperative anticoagulation and the level of the operation time, LAD, LVEF, left ventricular end-diastolic dimension (LVEDD), triglyceride (TG), total cholesterol (TC), Cr, CK-MB, TnI, $K^{+}$, ${\mathrm{Ca}}^{2+}$, ${\mathrm{Mg}}^{2+}$, and BNP (all *p* < 0.05) (as shown in **Supplementary Table 1**). Additionally, significant differences were present in age, the prevalence of CKD, prior percutaneous coronary intervention (PCI), the rate of on-pump CABG, postoperative anticoagulation and the level of operation time, LAD, LVEF, LVEDD, Cr, $K^{+}$, ${\mathrm{Ca}}^{2+}$, ${\mathrm{Mg}}^{2+}$, and BNP (all *p* < 0.05) between the groups with POAF durations shorter than 48 hours and the group with POAF durations longer than 48 hours (Table 1). After applying PSM and IPTW, the standardized mean difference for all baseline variables was <0.1, indicating that the baseline characteristics between groups were balanced (**Supplementary Figs. 1,2**).

**Table 1.** **Baseline characteristics between the POAF durations shorter than 48 hours and POAF durations longer than 48 hours groups**.

	Unmatched			1:1 PSM			IPTW		
	POAF <48 h	POAF ≥48 h	*p* value	POAF <48 h	POAF ≥48 h	*p* value	POAF <48 h	POAF ≥48 h	*p* value
n	2473	1131		1098	1098		1093.06	1096.53	
Age, years	65 (59, 70)	66 (60, 71)	<0.001*	66 (60, 71)	66 (60, 71)	0.724	66 (60, 71)	66 (60, 71)	0.996
Male (%)	1930 (78)	898 (79.4)	0.381	862 (78.5)	876 (79.8)	0.495	855.3 (78.3)	874 (79.7)	0.336
BMI, kg/$m^{2}$	25.8 (23.9, 27.5)	25.8 (24.2, 27.6)	0.367	25.8 (24.1, 27.4)	25.8 (24.2, 27.6)	0.424	25.8 (23.9, 27.4)	25.8 (24.2, 27.6)	0.236
Hypertension (%)	1580 (63.9)	708 (62.6)	0.478	711 (64.8)	685 (62.4)	0.268	696.7 (63.7)	685.8 (62.5)	0.5
Diabetes (%)	954 (38.6)	466 (41.2)	0.144	405 (36.9)	449 (40.9)	0.06	424.2 (38.8)	448.6 (40.9)	0.246
COPD (%)	61 (2.5)	31 (2.7)	0.711	25 (2.3)	28 (2.6)	0.89	28.6 (2.6)	28.4 (2.6)	0.973
Hyperlipidemia (%)	1429 (57.8)	630 (55.7)	0.256	625 (56.9)	616 (56.1)	0.731	628.9 (57.5)	612.9 (55.9)	0.369
CKD (%)	66 (2.7)	54 (4.8)	0.002*	43 (3.9)	49 (4.5)	0.594	44.4 (4.1)	45.2 (4.1)	0.944
PCI history (%)	247 (10)	143 (12.6)	0.02*	140 (12.8)	136 (12.4)	0.847	134.6 (12.3)	136 (12.4)	0.946
Stroke history (%)	389 (15.7)	184 (16.3)	0.718	177 (16.1)	179 (16.3)	0.954	178.9 (16.4)	178.9 (16.3)	0.971
Cardiac surgery history (%)	29 (1.2)	12 (1.1)	0.901	16 (1.5)	11 (1.0)	0.439	11.8 (1.1)	11.6 (1.1)	0.962
On-pump CABG (%)	197 (8)	138 (12.2)	<0.001*	122 (11.1)	121 (11.0)	1	123.7 (11.3)	123.8 (11.3)	0.982
Operation time, h	4 (4, 5)	4 (4, 5)	0.009*	4 (4, 5)	4 (4, 5)	0.782	4 (4, 5)	4 (4, 5)	0.788
Anticoagulation (%)	48 (1.9)	38 (3.4)	0.013*	26 (2.4)	38 (3.5)	0.164	26.1 (2.4)	36.8 (3.4)	0.13
Antiplatelet (%)	2400 (97.1)	1094 (96.7)	0.632	1066 (97.2)	1068 (97.3)	0.997	1054.9 (96.6)	1064.3 (97.1)	0.485
Electrical cardioversion (%)	135 (5.5)	68 (6.0)	0.504	61 (5.6)	65 (5.9)	0.714	60.1 (5.5)	63.8 (5.8)	0.727
POAF duration (h)	16 (8, 21)	74 (60, 89)	<0.001*	16 (8, 21)	74 (60, 89)	<0.001*	16 (8, 21)	74 (60, 89)	<0.001*
Preoperative laboratory data									
TG, mmol/L	1.6 (1.15, 1.85)	1.52 (1.07, 1.78)	<0.001*	1.56 (1.08, 1.78)	1.52 (1.07, 1.78)	0.333	1.51 (1.07, 1.76)	1.50 (1.05, 1.77)	0.608
TC, mmol/L	3.96 (3.38, 4.35)	3.96 (3.31, 4.3)	<0.001*	3.96 (3.29, 4.21)	3.94 (3.26, 4.26)	0.975	3.96 (3.31, 4.28)	3.94 (3.26, 4.26)	0.469
Cr, µmol/L	69 (59, 81.1)	73 (61.8, 86.8)	<0.001*	74.7 (62.3, 88.55)	74.7 (63.5, 90.88)	0.23	74.3 (62.12, 87.89)	74.62 (63.25, 90.75)	0.112
UA, µmol/L	334.2 (287.2, 376.1)	334.2 (282.6, 381.2)	0.747	334.2 (289.9, 382.8)	334.2 (282.5, 380.3)	0.37	334.2 (289.5, 379.7)	334.2 (282.5, 380.8)	0.426
$K^{+}$, mmol/L	4.08 (3.83, 4.32)	4.1 (3.87, 4.39)	0.002*	4.1 (3.87, 4.38)	4.1 (3.86, 4.38)	0.768	4.09 (3.85, 4.36)	4.1 (3.86, 4.38)	0.605
${\mathrm{Ca}}^{2+}$, mmol/L	2.21 (2.04, 2.33)	2.2 (2.03, 2.31)	0.01*	2.19 (2.01, 2.31)	2.2 (2.03, 2.31)	0.636	2.19 (2.02, 2.31)	2.2 (2.03, 2.31)	0.934
${\mathrm{Mg}}^{2+}$, mmol/L	0.88 (0.82, 0.94)	0.89 (0.84, 0.95)	0.001*	0.89 (0.83, 0.95)	0.89 (0.84, 0.95)	0.747	0.89 (0.83, 0.95)	0.89 (0.84, 0.95)	0.827
CK-MB, ng/mL	3.1 (1.7, 6.4)	3.1 (1.7, 6.8)	0.469	3.3 (1.9, 7.2)	3 (1.7, 6.8)	0.115	3.3 (1.8, 7.1)	3 (1.7, 6.74)	0.159
TnI, pg/mL	0.18 (0.07, 0.51)	0.2 (0.06, 0.64)	0.086	0.19 (0.07, 0.58)	0.19 (0.06, 0.62)	0.978	0.2 (0.07, 0.61)	0.19 (0.06, 0.61)	0.688
Mb, ng/mL	176.9 (40.7, 303)	175.7 (40.5, 306.9)	0.991	184.9 (45.5, 312.5)	173.9 (38.9, 302.8)	0.222	181.7 (44.3, 310.1)	175.4 (39.5, 306)	0.436
BNP, pg/mL	205 (87, 338)	265 (105.5, 474.5)	<0.001*	242 (106.25, 410)	257 (103, 451)	0.361	248 (109, 414.26)	255 (103, 450)	0.697
Preoperative echocardiographic data									
LAD, mm	36.66 (35, 38)	36.66 (35, 39)	<0.001*	36.66 (36, 40)	36.66 (36, 40)	0.379	36.66 (36, 40)	36.66 (36, 40)	0.282
LVEF, %	59.43 (58, 65)	59.43 (56, 63)	<0.001*	59.43 (55, 63)	59.43 (55, 63)	0.984	59.43 (55, 63)	59.43 (55, 63)	0.569
E/A ratio	0.84 (0.68, 0.85)	0.85 (0.67, 0.85)	0.454	0.85 (0.66, 0.85)	0.85 (0.66, 0.85)	0.637	0.83 (0.67, 0.85)	0.85 (0.66, 0.85)	0.661
LVEDD, mm	32.34 (29, 33)	32.34 (30, 34)	<0.001*	32.34 (30, 35)	32.34 (30, 35)	0.66	32.34 (30, 35)	32.34 (30, 35)	0.551

Data are presented as median (25th–75th percentiles) or n (%). *, there were significant differences between the POAF <48 h patients and POAF ≥48 h patients. BMI, body mass index; COPD, chronic obstructive pulmonary disease; CKD, chronic kidney disease; PCI, percutaneous coronary intervention; TG, triglyceride; TC, total cholesterol; Cr, creatinine; UA, uric acid; CK-MB, creatine kinase MB; TnI, troponin I; Mb, myoglobin; BNP, brain natriuretic peptide; LAD, left atrial diameter; LVEF, left ventricular ejection fractions; E/A, ratio early to late diastolic transmitral flow velocity; LVEDD, left ventricular end-diastolic dimension; POAF, postoperative atrial fibrillation; PSM, propensity score matching; IPTW, inverse probability of treatment weighting; CABG, coronary artery bypass grafting.

### 3.2 Comparing Primary and Secondary Outcomes between Non-POAF and POAF

We compared the primary outcomes between the non-POAF and POAF groups and found that after applying PSM and IPTW adjustments the in-hospital mortality, ARF, AKI, and significant GIB were all significantly higher among POAF patients than among non-POAF patients (**Supplementary Table 2**). These outcomes were determined to be independently associated with POAF by logistic regression analyses (**Supplementary Table 3**). However, even though the stroke incidence was higher among POAF compared to non-POAF, no statistically significant difference was present, nor was stroke occurrence associated with POAF in the logistic regression analyses. In terms of secondary outcomes, we also found that POAF patients had longer LOS and ICU durations.

### 3.3 Comparing Primary and Secondary Outcomes between Patients with POAF Durations Longer than 48 Hours and Patients with POAF Durations Shorter than 48 Hours

To determine whether POAF duration affected the prognosis of postoperative CABG patients, we compared the primary outcomes between the groups with POAF durations longer than 48 hours and POAF durations shorter than 48 hours. For the primary outcomes and prior to applying PSM and IPTW adjustments, we found no significant difference in the incidence of stroke (1.9% vs. 1.3%, *p* = 0.147) in patients with POAF durations longer than 48 hours compared to the controls. However, there were higher rates of in-hospital mortality, ARF, AKI, and significant GIB in the patients with POAF durations longer than 48 hours (all *p* < 0.001). The lack of any significant differences between the two groups was also observed in stroke occurrence after adjusting for PSM and IPTW (PSM 2.0% vs. 1.3%, *p* = 0.239; IPTW 1.9% vs. 1.4%, *p* = 0.275), as well as for in-hospital mortality (PSM 3.1% vs. 2.5%, *p* = 0.436; IPTW 3.3% vs. 2.5%, *p* = 0.301); however, significant differences were still present for the other outcomes (Table 2).

**Table 2.** **Primary and secondary outcomes between POAF durations shorter than 48 hours and POAF durations longer than 48 hours groups**.

	Unadjusted			Matched			Weighted		
	POAF <48 h	POAF ≥48 h	*p* value	POAF <48 h	POAF ≥48 h	*p* value	POAF <48 h	POAF ≥48 h	*p* value
In-hospital mortality (%)	38 (1.5)	41 (3.6)	<0.001*	27 (2.5)	34 (3.1)	0.436	27.7 (2.5)	35.3 (3.3)	0.301
Stroke (%)	31 (1.3)	22 (1.9)	0.147	14 (1.3)	22 (2.0)	0.239	15.5 (1.4)	21.3 (1.9)	0.275
ARF (%)	14 (0.6)	21 (1.9)	<0.001*	6 (0.5)	20 (1.8)	0.01*	7.9 (0.7)	19.6 (1.8)	0.011*
AKI (%)	35 (1.4)	51 (4.5)	<0.001*	25 (2.3)	42 (3.8)	0.047*	24.9 (2.3)	40.8 (3.7)	0.032*
Significant GIB (%)	17 (0.7)	27 (2.4)	<0.001*	10 (0.9)	25 (2.3)	0.017*	11.2 (1.0)	23.1 (2.1)	0.023*
Postoperative LOS, days	7 (6, 9)	8 (7, 12)	<0.001*	7 (6, 9)	8 (7, 12)	<0.001*	7 (6, 9)	8 (7, 12)	<0.001*
ICU duration, hours	21 (17, 61)	27 (19.8, 89.5)	<0.001*	21.9 (17, 65)	26.4 (19.8, 87.8)	<0.001*	21.8 (17.8, 65.94)	26 (19.8, 87.8)	<0.001*

Data are presented as median (25th–75th percentiles) or n (%). *, there were significant differences between the POAF <48 h patients and POAF ≥48 h patients. ARF, acute respiratory failure; AKI, acute kidney failure; GIB, gastrointestinal bleeding; LOS, length of stay; ICU, intensive care unit; POAF, postoperative atrial fibrillation.

**Fig. 2.**
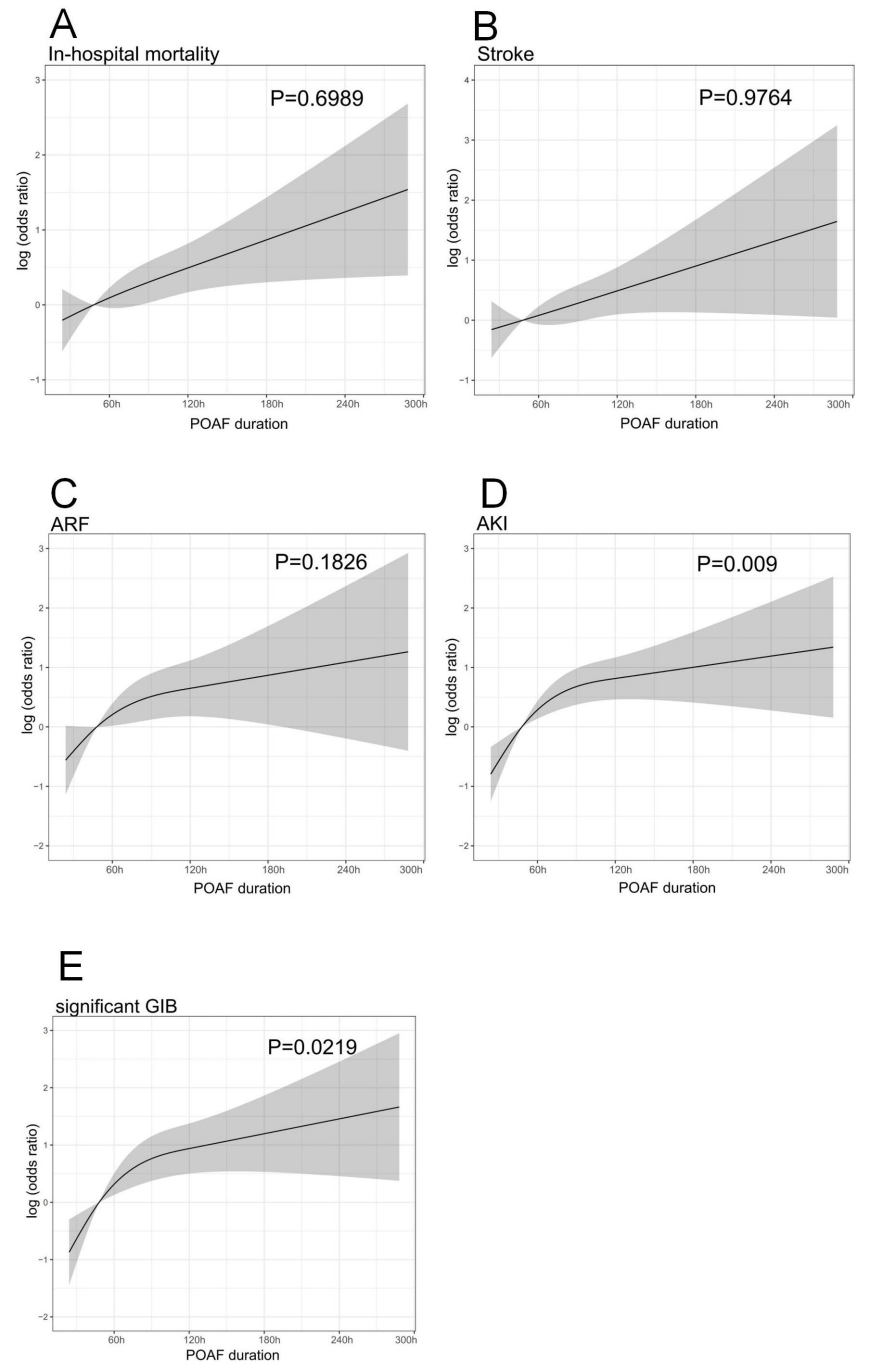
**Restricted cubic spline plots of associations between POAF duration and postoperative complications**. (A) In-hospital mortality, (B) stroke, (C) acute respiratory failure (ARF), (D) acute kidney injury (AKI), (E) significant gastrointestinal bleeding (GIB). The solid line and shaded area represent the log-transformed odds ratios and corresponding 95% confidence intervals. A linear relationship was present between POAF duration with in-hospital mortality, stroke, and ARF (all *p* for non-linearity >0.05). By contrast, a non-linear relationship was found between POAF duration and AKI (*p* for non-linearity = 0.009) and significant GIB (*p* for non-linearity = 0.0219). POAF, postoperative atrial fibrillation.

To further elucidate the relationship between POAF duration and the primary outcomes, RCS analyses were conducted, whereby a linear relationship was present between POAF duration and in-hospital mortality, stroke, and ARF (all *p* for nonlinearity >0.05). In contrast, a nonlinear relationship was found between POAF duration and AKI (*p* for nonlinearity = 0.009) and significant GIB (*p* for nonlinearity = 0.0219), for which the inflection point was 49 hours, indicating that the incidences of AKI and significant GIB increased after POAF durations >49 hours (Fig. 2).

We also conducted logistic regression analyses, in which a longer POAF duration was not independently associated with in-hospital death (adjusted OR: 1.60, 95% CI: 0.97–2.65, *p* = 0.068) and stroke (adjusted OR: 1.28, 95% CI: 0.71–2.34, *p* = 0.414) but was independently associated with ARF (adjusted OR: 2.96, 95% CI: 1.47–6.09, *p* = 0.003), AKI (adjusted OR: 2.37, 95% CI: 1.42–3.99, *p* = 0.001), and significant GIB (adjusted OR: 2.60, 95% CI: 1.38–5.03, *p* = 0.004). Moreover, the results were still valid even after adjusting for PSM and IPTW (Table 3). Furthermore, the POAF group with a duration longer than 48 hours had a longer postoperative LOS and ICU duration (Table 2).

**Table 3.** **The ORs for primary outcomes in patients with POAF longer than 48 hours**.

Methods	In-hospital mortality		Stroke		ARF		AKI		Significant GIB	
	OR (95%CI)	*p* value	OR (95%CI)	*p* value	OR (95%CI)	*p* value	OR (95%CI)	*p* value	OR (95%CI)	*p* value
Unadjusted	2.29 (1.46–3.58)	<0.001	1.56 (0.89–2.70)	0.112	3.32 (1.70–6.70)	0.001	3.29 (2.14–5.12)	<0.001	3.53 (1.94–6.63)	<0.001
Matched	1.27 (0.76–2.13)	0.364	1.58 (0.81–3.18)	0.182	3.38 (1.43–9.26)	0.009	1.71 (1.04–2.86)	0.037	2.53 (1.25–5.56)	0.014
Weighted	1.28 (0.87–1.91)	0.218	1.38 (0.83–2.33)	0.220	2.49 (1.34–4.93)	0.006	1.66 (1.12–2.48)	0.012	2.08 (1.20–3.72)	0.011
Multivariable	1.60 (0.97–2.65)	0.068	1.28 (0.71–2.34)	0.414	2.96 (1.47–6.09)	0.003	2.37 (1.42–3.99)	0.001	2.60 (1.38–5.03)	0.004

ARF, acute respiratory failure; AKI, acute kidney failure; GIB, gastrointestinal bleeding; POAF, postoperative atrial fibrillation; OR. odds ratio.

To control the bias in the effect of anticoagulation and antiplatelet therapy on bleeding/ischemic events, we further included postoperative anticoagulation and antiplatelet therapy (only records of postoperative anticoagulation and antiplatelet regimens prior to stroke or significant GIB occurrences), applying the same confounding variables in RCS into the multivariable logistic regression of stroke and significant GIB. The association between prolonged POAF duration and stroke (adjusted OR: 1.23, 95% CI: 0.67–2.25, *p* = 0.497) and significant GIB (adjusted OR: 2.61, 95% CI: 1.38–5.05, *p* = 0.003) remained consistent with the previous multivariable logistic regression analyses (stroke: adjusted OR: 1.28, 95% CI: 0.71–2.34, *p* = 0.414; significant GIB: adjusted OR: 2.60, 95% CI: 1.38–5.03, *p* = 0.004), after including postoperative anticoagulation and antiplatelet therapy as confounding variables in the logistic regression analyses.

### 3.4 Risk Factor Analysis for Prolonged POAF Durations

After including POAF risk factors, such as age, male sex, BMI, hypertension, diabetes, LAD, LVEF, CK-MB, $K^{+}$, and BNP, in the multivariable logistic regression analysis, we found that the statistically significant risk factors were age, BMI, on-pump CABG, long operation time, ${\mathrm{Mg}}^{2+}$, CK-MB, TnI, BNP, LAD, and low LVEF (all *p* < 0.05, shown in **Supplementary Table 4**).

## 4. Discussion

POAF is a common complication after CABG; however, the impact of POAF duration on the occurrence of other postsurgical complications has remained largely unexamined. To the best of our knowledge, this is the first study to focus on the impact of prolonged POAF duration on perioperative outcomes among CABG patients. Our findings suggest that POAF is associated with increased in-hospital mortality, ARF, AKI, and significant GIB compared to non-POAF. Additionally, POAF durations longer than 48 hours were independently associated with postoperative ARF, AKI, and significant GIB but not with in-hospital mortality or stroke. Patients with POAF durations ≥48 hours had longer postoperative ICU durations and LOS. Moreover, all of these findings remained, even after applying PSM and IPTW adjustments to control for differences between the baseline characteristics in the non-POAF and POAF groups, as well as the POAF <48 hours and ≥48 hours patient groups.

Many studies have confirmed the correlation between POAF and in-hospital mortality and many adverse perioperative outcomes, such as perioperative stroke and perioperative acute renal failure [4, 5], which is consistent with our observation. The long-term follow-up results showed that POAF is significantly associated with the development of subsequent AF [14, 15] and an adverse long-term prognosis [4, 16, 17, 18]. It is generally recognized that POAF possesses a brief and self-limited time course [19]. However, some studies have shown that not all POAF will disappear in a short time [6, 20]. Although limited studies have focused on the impact of the course of POAF on prognosis, which has led to clinicians having blind spots in the treatment of patients with POAF with delayed reversion to SR. Previous studies have shown that postoperative POAF durations longer than 48 hours or POAF recurrent events more than twice after cardiac surgery are independently associated with reduced long-term survival [7, 21]. Rezk *et al*. [22] showed that POAF patients with electrical cardioversion or sustained AF were independently associated with an increased long-term risk of heart failure but not with an increased long-term risk of death, thromboembolic complications, or bleeding. These results of poor prognosis show that we should not only focus on the occurrence or absence of POAF but also regard them as a ”continuous variable” to project further analysis.

However, these studies mostly focused on long-term prognoses, which may have the disadvantage of the underlying reasons behind their differences being obscured by the accumulation of confounding factors over time. Although this disadvantage is mitigated in our study by focusing on short-term perioperative prognoses, the greater capacity to identify underlying risk factors for adverse events is more critical for perioperative cardiac surgical management. This is due to this stage being associated with the highest postoperative mortality and complication occurrence rates. Thus, minimizing perioperative adverse event risk is a crucial concern. Although Sigurdsson *et al*. [7] mentioned that prolonged POAF durations increased postoperative LOS and ICU duration in patients undergoing cardiac surgery, the study did not further analyze other perioperative endpoints. In the analysis of in-hospital outcomes by patients with prolonged POAF durations, a study of ICU patients, excluding cardiac surgery, showed that patients with new-onset AF had no significant difference or in-hospital death or stroke compared to the control, although there was a time-dependent association between AF duration and hospital mortality [23]. One study also highlighted that new-onset AF lasting more than 6 hours in ICU patients is associated with in-hospital death and stroke [24].

Anticoagulation regimens lasting for at least 3 weeks prior to and 4 weeks after cardioversion have long been recommended for AF durations longer than 48 hours to reduce stroke and systemic embolism risks [9]. Although anticoagulation of POAF is recommended in all guidelines, the class of recommendation varies. The American Heart Association/American College of Cardiology/Heart Rhythm Society (AHA/ACC/HRS) and European Society of Cardiology (ESC) guidelines do not specify a threshold duration time for initiating treatment [25, 26], whereas the Canadian Cardiovascular Society (CCS) recommends anticoagulation for POAF lasting more than 72 hours [27], whereas the same time point is 48 hours in the European Association for Cardio-Thoracic Surgery (EACTS) guidelines [28]. Furthermore, there is a lack of convincing evidence to support anticoagulation at these time points in POAF. This is not helped by the presence of polarized results regarding the usage of anticoagulation regimens for POAF patients, in which multiple studies have indicated that such patients do not benefit from these regimens due to increased bleeding risk [29, 30]. This makes the anticoagulation regimens of POAF controversial in clinical practice, in part because the effect of a prolonged duration of POAF on prognosis is unknown. In this study, we evaluated the effect of POAF lasting more than 48 hours on in-hospital stroke. Although the stroke incidence was higher in patients with POAF durations longer than 48 hours, the difference was not statistically significant. Considering the high risk of anticoagulation-related bleeding, we recommend that the benefits of routinely starting anticoagulation after POAF should be carefully weighed, particularly among those patients with low CHA2DS2-VASc scores.

Despite the current focus on the risk of thromboembolism and the indication of anticoagulation therapy for prolonged POAF, other perioperative problems caused by prolonged POAF leave much room for further exploration. We found that prolonged POAF duration is independently associated with perioperative ARF, AKI, and significant GIB, thereby suggesting that it was a noteworthy factor behind poorer perioperative outcomes among CABG patients. We speculate that persistent POAF may cause a prolonged period of reduced cardiac ejection, which causes a continuous worsening of cardiac function that causes the blood pressure to lower, making the perfusion of peripheral organs insufficient. Prolonged POAF may be associated with higher sympathetic excitation, which causes oxygen depletion and metabolic abnormalities in the body. In addition, prolonged POAF may increase the level of inflammatory response in the body, and the release of large amounts of harmful inflammatory factors may increase tissue damage and cause organ dysfunction. However, the above speculations need to be further verified by experiments.

Based on these findings and explorations, we suggest that the detection of POAF should be intensified and that once POAF is detected, proactive electrical cardioversion may be beneficial in improving the patient’s prognosis. In addition, several recent studies focusing on improved surgical approaches and intraoperative management to reduce the incidence of POAF have shown promising results, such as a reduction in retained blood into the pericardial sac and the use of Del Nido cardioplegia, which may represent a new direction in the treatment of POAF [31, 32, 33].

## 5. Conclusions

We found that POAF occurrence among CABG patients was associated with increased in-hospital mortality, ARF, AKI, significant GIB, and longer postoperative LOS and ICU durations. All of these patient outcomes, except for increased in-hospital mortality and stroke rate, were also linked to a POAF duration longer than 48 hours, compared to a duration shorter than 48 hours. Overall, the occurrence of poorer patient outcomes was higher among patients with longer POAF; therefore, postoperative monitoring of POAF and positive intervention after detection may be more helpful in optimizing post-CABG patient outcomes.

## 6. Limitation

In terms of monitoring POAF, not every patient was monitored until discharge, which leaves a proportion of patients with delayed onset of POAF who may not have been identified. The STS database definition of POAF has now been updated, and due to the early design of this study, the new criteria were not used to diagnose patients, which may have caused an overestimation in the number of patients with POAF. Given the intermittent nature of some POAF cases and its reoccurrence after conversion of the sinus rate with antiarrhythmic drugs, the exact duration of POAF could not be clearly recorded; therefore, the study design referring to previous studies used the time of last occurrence minus the time of first occurrence as the duration of POAF, which may lead to an overestimation of the duration. Limited by the characteristics of retrospective studies, some important variables, such as the use of postoperative vasoactive drugs and intraoperative variables were not counted in this study; however, studying the above indicators may provide new insights to explain the relationship between POAF and clinical outcomes.

## Data Availability

The datasets generated and/or analyzed during the current study are not publicly available due to the nature of this research, participants of this study did not agree for their data to be shared publicly but are available from the corresponding author on reasonable request.

## Supplementary Material

Supplementary material associated with this article can be found, in the online version, at https://doi.org/10.31083/j.rcm2503098.
