# Supplementary material for: Association of Postoperative Atrial Fibrillation Duration after Coronary Artery Bypass Grafting with Poor Postoperative Outcomes
Source: Rev Cardiovasc Med. 2024 Mar 8;25(3):98. doi: 10.31083/j.rcm2503098 (PMC11263830; doi:10.31083/j.rcm2503098)
Supplement: Supplementary file 1 [file 2153-8174-25-3-098-s1.zip › Supplementary material.docx]

**Supplementary Tables：**

**Supplementary Table 1. Baseline characteristics between POAF group and non-POAF group**

|  | **Unmatched** | | | **1:1 PSM** | | | **IPTW** | | |
| --- | --- | --- | --- | --- | --- | --- | --- | --- | --- |
|  | **non-POAF** | **POAF** | ***p value*** | **non-POAF** | **POAF** | ***p value*** | **non-POAF** | **POAF** | ***p value*** |
| **n** | 8244 | 3604 |  | 3506 | 3506 |  | 3478.27 | 3494.7 |  |
| **Age, years** | 62[55, 67] | 65[59, 70] | <0.001* | 65[59, 70] | 65.00[59, 70] | 0.698 | 65[59, 70] | 65[59, 70] | 0.971 |
| **Male (%)** | 6135 (74.4) | 2828 (78.5) | <0.001* | 2768 (79.0) | 2747 (78.4) | 0.56 | 2723.5 (78.3) | 2736.7 (78.3) | 0.991 |
| **BMI, kg/m^2^** | 25.8[23.8, 27.5] | 25.8[24, 27.6] | 0.17 | 25.8[23.8, 27.5] | 25.8[24, 27.6] | 0.105 | 25.7[23.8, 27.4] | 25.8[24, 27.6] | 0.007* |
| **Hypertension (%)** | 4988 (60.5) | 2288 (63.5) | 0.002* | 2258 (64.4) | 2214 (63.1) | 0.285 | 2208.3 (63.5) | 2216.2 (63.4) | 0.943 |
| **Diabetes (%)** | 3189 (38.7) | 1420 (39.4) | 0.473 | 1361 (38.8) | 1376 (39.2) | 0.732 | 1363.6 (39.2) | 1373.7 (39.3) | 0.917 |
| **COPD (%)** | 123 (1.5) | 92 (2.6) | <0.001* | 81 (2.3) | 83 (2.4) | 0.937 | 82.1 (2.4) | 81.5 (2.3) | 0.933 |
| **Hyperlipidemia (%)** | 4872 (59.1) | 2059 (57.1) | 0.048* | 2041 (58.2) | 2020 (57.6) | 0.629 | 2002.4 (57.6) | 2007.2 (57.4) | 0.899 |
| **CKD (%)** | 132 (1.6) | 120 (3.3) | <0.001* | 93 (2.7) | 93 (2.7) | 1 | 92.8 (2.7) | 96.9 (2.8) | 0.775 |
| **PCI history (%)** | 878 (10.7) | 390 (10.8) | 0.807 | 368 (10.5) | 372 (10.6) | 0.907 | 367.9 (10.6) | 376.0 (10.8) | 0.776 |
| **Stroke history (%)** | 1034 (12.5) | 573 (15.9) | <0.001* | 543 (15.5) | 544 (15.5) | 1 | 544.9 (15.7) | 545.4 (15.6) | 0.939 |
| **Cardiac surgery history (%)** | 37 (0.4) | 41 (1.1) | <0.001* | 33 (0.9) | 35 (1.0) | 0.903 | 32 (0.9) | 32.6 (0.9) | 0.953 |
| **On-pump CABG (%)** | 515 (6.2) | 335 (9.3) | <0.001* | 318 (9.1) | 302 (8.6) | 0.528 | 299.0 (8.6) | 299.9 (8.6) | 0.979 |
| **Operation time, h** | 4[4, 5] | 4[4, 5] | <0.001* | 4[4, 5] | 4[4, 5] | 0.308 | 4[4, 5] | 4[4, 5] | 0.683 |
| **Anticoagulation (%)** | 83 (1.0) | 86 (2.4) | <0.001* | 39 (1.1) | 83 (2.4) | <0.001* | 39.2 (1.1) | 81.9 (2.3) | <0.001* |
| **Antiplatelet (%)** | 8035 (97.5) | 3494 (97.0) | 0.099 | 3407 (97.3) | 3415 (97.4) | 0.707 | 3375.8 (97.1) | 3395.7 (97.2) | 0.832 |
| **Preoperative laboratory data** | | | | | | | | | |
| **TG, mmol/L** | 1.60[1.15, 1.85] | 1.52[1.07, 1.78] | <0.001* | 1.54[1.1, 1.76] | 1.52[1.07, 1.79] | 0.42 | 1.54[1.11, 1.76] | 1.52[1.07, 1.79] | 0.218 |
| **TC, mmol/L** | 3.96[3.38, 4.35] | 3.96[3.31, 4.3] | <0.001* | 3.96[3.33, 4.27] | 3.96[3.31, 4.3] | 0.808 | 3.96[3.34, 4.28] | 3.96[3.31, 4.3] | 0.35 |
| **Cr, μmol/L** | 69[59, 81.1] | 73[61.8, 86.8] | <0.001* | 71.5[60.8, 83.97] | 72.6[61.6, 86.2] | 0.01* | 71.3[60.8, 83.9] | 72.5[61.6, 86.1] | 0.001* |
| **UA, μmol/L** | 334.2[280.9, 371.3] | 334.2[286, 377.9] | 0.064 | 334.2[284.2, 372.1] | 334.2[284.5, 377.9] | 0.4 | 334.2[283.4, 375.4] | 334.2[284.9, 376.8] | 0.865 |
| **K^+^, mmol/L** | 4.07[3.84, 4.29] | 4.09[3.84, 4.34] | <0.001* | 4.09[3.85, 4.33] | 4.08[3.84, 4.33] | 0.739 | 4.09[3.85, 4.32] | 4.09[3.84, 4.33] | 0.974 |
| **Ca^2+^, mmol/L** | 2.22[2.06, 2.33] | 2.21[2.04, 2.32] | 0.004* | 2.21[2.04, 2.32] | 2.21[2.04, 2.32] | 0.796 | 2.21[2.04, 2.32] | 2.21[2.04, 2.32] | 0.856 |
| **Mg^2+^, mmol/L** | 0.88[0.82, 0.93] | 0.88[0.83, 0.95] | <0.001* | 0.88[0.83, 0.94] | 0.88[0.83, 0.94] | 0.803 | 0.88[0.82, 0.94] | 0.88[0.83, 0.94] | 0.941 |
| **CK-MB, ng/ml** | 2.9 [1.7, 6.1] | 3.1[1.7, 6.6] | 0.004* | 3.3[1.8, 6.9] | 3[1.7, 6.4] | 0.026* | 3.1[1.8, 6.7] | 3.1[1.7, 6.4] | 0.292 |
| **TnI, pg/ml** | 0.18[0.07, 0.49] | 0.19[0.07, 0.54] | 0.049* | 0.19[0.07, 0.57] | 0.18[0.07, 0.52] | 0.138 | 0.19[0.07, 0.57] | 0.18[0.07, 0.52] | 0.247 |
| **Mb, ng/ml** | 176[40.4, 315] | 176.45[40.7, 304.62] | 0.626 | 181.25[39.73, 316.75] | 174.05[39.9, 302] | 0.227 | 177.11[40.1, 315] | 175.52[39.91, 304] | 0.433 |
| **BNP, pg/ml** | 175[74, 276] | 220[92, 377] | <0.001* | 209[93, 345] | 213[90, 362] | 0.446 | 209[91, 336] | 212.28[89, 358] | 0.317 |
| **Preoperative echocardiographic data** | | | | | | | | | |
| **LAD, mm** | 36.66[35, 38] | 36.66[35, 39] | <0.001* | 36.66[35, 39] | 36.66[35, 39] | 0.923 | 36.66[35, 39] | 36.66[35, 39] | 0.979 |
| **LVEF, %** | 59.43[58, 65] | 59.43[56, 63] | <0.001* | 59.43[56, 64] | 59.43[56, 64] | 0.904 | 59.43[56, 64] | 59.43[56, 64] | 0.86 |
| **E/A ratio** | 0.84[0.68, 0.85] | 0.85[0.67, 0.85] | 0.454 | 0.81[0.65, 0.85] | 0.85[0.67, 0.85] | 0.001* | 0.81[0.66, 0.85] | 0.85[0.67, 0.85] | 0.002* |
| **LVEDD, mm** | 32.34[29, 33] | 32.34[30, 34] | <0.001* | 32.34[30, 34] | 32.34[30, 34] | 0.617 | 32.34[30, 34] | 32.34[30, 34] | 0.616 |

Data are presented as median [25th -75th percentiles] or n (%). *, there were significant differences between the non-POAF patients and POAF patients. BMI, body mass index; COPD, chronic obstructive pulmonary disease; CKD, chronic kidney disease; PCI, percutaneous coronary intervention; TG, triglyceride; TC, total cholesterol; Cr, creatinine; UA, uric acid; CK-MB, creatine kinase MB; TnI, troponin I; Mb, myoglobin; BNP, brain natriuretic peptide; LAD, left atrial diameter; LVEF, left ventricular ejection fractions; E/A, ratio early to late diastolic transmitral flow velocity; LVEDD, left ventricular end-diastolic dimension

**Supplementary Table 2. Primary and secondary outcomes between non-POAF group and POAF group**

|  | **Unadjusted** | | | **Matched** | | | **Weighted** | | |
| --- | --- | --- | --- | --- | --- | --- | --- | --- | --- |
|  | **non-POAF** | **POAF** | ***p value*** | **non-POAF** | **POAF** | ***p value*** | **non-POAF** | **POAF** | ***p value*** |
| **In-hospital mortality (%)** | 46 (0.6) | 79 (2.2) | <0.001* | 31 (0.9) | 59 (1.7) | 0.004* | 32.7 (0.9) | 62.1 (1.8) | 0.001* |
| **Stroke (%)** | 74 (0.9) | 56 (1.5) | 0.002* | 35 (1.0) | 49 (1.4) | 0.153 | 37.6 (1.1) | 50.6 (1.4) | 0.114 |
| **ARF (%)** | 26 (0.3) | 40 (1.1) | <0.001* | 9 (0.3) | 39 (1.1) | <0.001* | 13.8 (0.4) | 36.1 (1.0) | <0.001* |
| **AKI (%)** | 30 (0.4) | 93 (2.6) | <0.001* | 20 (0.6) | 72 (2.1) | <0.001* | 22.4 (0.6) | 69.7 (2.0) | <0.001* |
| **Significant GIB (%)** | 22 (0.3) | 46 (1.3) | <0.001* | 13 (0.4) | 39 (1.1) | 0.001* | 13.9 (0.4) | 37.6 (1.1) | <0.001* |
| **Postoperative LOS, days** | 7[6, 8] | 7 [6, 10] | <0.001* | 7 [6, 8] | 7 [6, 10] | <0.001* | 7 [6, 8] | 7 [6, 10] | <0.001* |
| **ICU stay, hours** | 20.8 [16, 25.8] | 22 [17, 67.85] | <0.001* | 21 [16.8, 27.8] | 21.8 [17, 66.8] | <0.001* | 21 [16.8, 27] | 21.8 [17, 66] | <0.001* |

Data are presented as median [25th -75th percentiles] or n (%). *, there were significant differences between the non-POAF patients and POAF patients. ARF, acute respiratory failure; AKI, acute kidney failure; GIB, gastrointestinal bleeding; LOS, length of stay; ICU, intensive care unit

**Supplementary Table 3. The OR of primary outcomes for POAF group**

| **Methods** | **In-hospital mortality** | | **Stroke** | | **ARF** | | **AKI** | | **Significant GIB** | |
| --- | --- | --- | --- | --- | --- | --- | --- | --- | --- | --- |
|  | ***OR***  ***(95%CI)*** | ***p value*** | ***OR (95%CI)*** | ***p value*** | ***OR (95%CI)*** | ***p value*** | ***OR (95%CI)*** | ***p value*** | ***OR (95%CI)*** | ***p value*** |
| **Unadjusted** | 3.99 (2.78-5.80) | <0.001 | 1.74 (1.22-2.47) | 0.002 | 3.55 (2.17-5.89) | <0.001 | 7.25 (4.86-11.14) | <0.001 | 4.83 (2.94-8.19) | <0.001 |
| **Matched** | 1.74 (1.14-2.70) | 0.012 | 1.54 (0.99-2.43) | 0.059 | 4.37 (2.21-9.64) | <0.001 | 3.65 (2.27-6.17) | <0.001 | 3.02 (1.66-5.89) | 0.001 |
| **Weighted** | 1.91 (1.38-2.66) | <0.001 | 1.35 (0.97-1.87) | 0.075 | 2.62 (1.65-4.31) | <0.001 | 3.13 (2.19-4.58) | <0.001 | 2.71 (1.72-4.44) | <0.001 |
| **Multivariable** | 2.53 (1.70-3.81) | <0.001 | 1.28 (0.88-1.85) | 0.195 | 2.93 (1.76-4.95) | <0.001 | 5.41 (3.46-8.70) | <0.001 | 3.45 (2.04-5.98) | <0.001 |

ARF, acute respiratory failure; AKI, acute kidney failure; GIB, gastrointestinal bleeding

**Supplementary Table 4. Logistic regression analysis for the prognosis of POAF duration longer than 48 hours.**

| **Risk factor** | **Univariate analysis** | | |  | **Multivariate analysis** | | |
| --- | --- | --- | --- | --- | --- | --- | --- |
|  | **OR** | **95%CI** | ***P value*** |  | **OR** | **95%CI** | ***P value*** |
| **Age** | 1.018 | 1.009-1.027 | <0.001 |  | **1.022** | **1.013-1.031** | **<0.001** |
| **Male** | 1.084 | 0.912-1.289 | 0.358 |  | 1.148 | 0.957-1.379 | 0.138 |
| **BMI** | 1.019 | 0.996-1.043 | 0.111 |  | **1.029** | **1.004-1.005** | **0.025** |
| **Hypertension** | 0.946 | 0.818-1.094 | 0.455 |  | 0.924 | 0.794-1.077 | 0.313 |
| **Diabetes** | 1.116 | 0.967-1.288 | 1.288 |  | 1.092 | 0.940-1.268 | 0.250 |
| **COPD** | 1.114 | 0.719-1.727 | 0.628 |  | 1.025 | 0.655-1.605 | 0.912 |
| **Hyperlipidemia** | 0.919 | 0.797-1.059 | 0.241 |  | 0.951 | 0.822-1.100 | 0.501 |
| **CKD** | 1.829 | 1.267-2.639 | 0.001 |  | 1.259 | 0.850-1.864 | 0.250 |
| **PCI history** | 1.304 | 1.048-1.624 | 0.017 |  | 1.254 | 0.997-1.570 | 0.059 |
| **Stroke history** | 1.041 | 0.860-1.261 | 0.681 |  | 0.998 | 0.819-1.216 | 0.982 |
| **Cardiac surgery history** | 0.904 | 0.459-1.778 | 0.769 |  | 0.979 | 0.488-1.961 | 0.952 |
| **On-pump CABG** | 0.001 | 1.276-2.021 | <0.001 |  | **1.386** | **1.087-1.767** | **0.009** |
| **Operation time** | 0.010 | 1.015-1.122 | 0.01 |  | **1.053** | **1.000-1.109** | **0.049** |
| **TG** | 0.942 | 0.869-1.021 | 0.145 |  | 0.954 | 0.876-1.039 | 0.276 |
| **TC** | 0.948 | 0.878-1.023 | 0.168 |  | 0.969 | 0.895-1.049 | 0.436 |
| **Cr** | 1.004 | 1.002-1.006 | <0.001 |  | 1.001 | 0.999-1.003 | 0.178 |
| **UA** | 1.000 | 0.999-1.001 | 0.574 |  | 1.000 | 0.999-1.000 | 0.311 |
| **K^+^** | 1.305 | 1.103-1.545 | 0.002 |  | 1.180 | 0.992-1.403 | 0.061 |
| **Ca^2+^** | 0.657 | 0.459-0.940 | 0.021 |  | 0.781 | 0.540-1.130 | 0.190 |
| **Mg^2+^** | 4.13 | 2.246-7.595 | <0.001 |  | **2.891** | **1.532-5.456** | **0.001** |
| **CK-MB** | 1.003 | 1.001-1.006 | 0.010 |  | **1.002** | **1.000-1.005** | **0.049** |
| **TnI** | 1.031 | 1.015-1.048 | <0.001 |  | **1.020** | **1.002-1.039** | **0.028** |
| **Mb** | 1.000 | 1.000-1.000 | 0.994 |  | 1.000 | 1.000-1.000 | 0.742 |
| **BNP** | 1.001 | 1.000-1.001 | <0.001 |  | **1.001** | **1.000-1.005** | **<0.001** |
| **LAD** | 1.048 | 1.029-1.066 | <0.001 |  | **1.026** | **1.007-1.046** | **0.008** |
| **LVEF** | 0.973 | 0.965-0.982 | <0.001 |  | **0.984** | **0.874-0.993** | **0.001** |
| **E/A** | 1.020 | 0.819-1.269 | 0.862 |  | 1.031 | 0.822-1.292 | 0.793 |

Multivariate analysis was adjusted for age, male gender, BMI, hypertension, diabetes, LAD, LVEF, CK-MB, K+, BNP.

**Supplementary Figure：**

**Supplementary Fig. 1. Covariate balance plot for assessing balance between non-POAF and POAF groups after PSM and IPTW**


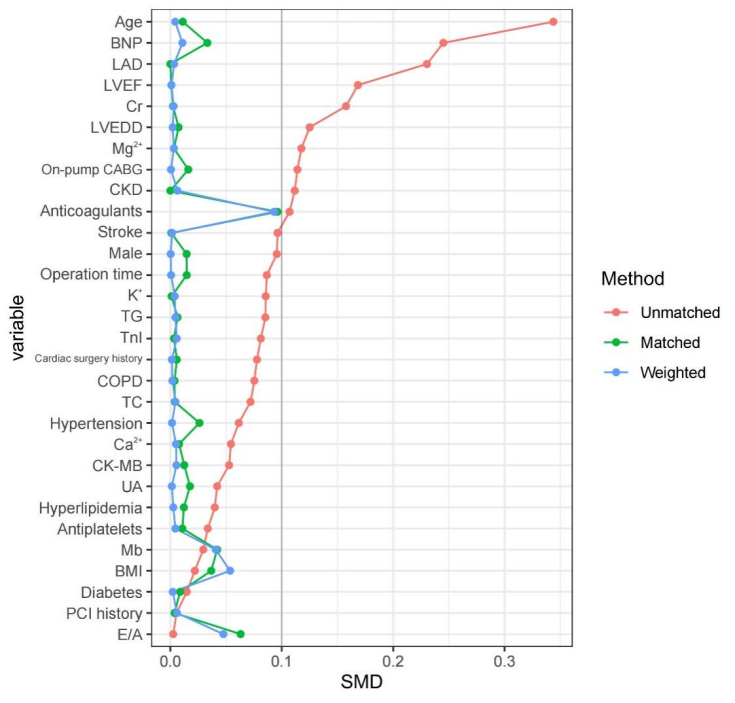


After using PSM with IPTW, the SMD for all variables between the non-POAF and POAF groups were <0.1.

SMD, standardized mean difference; BMI, body mass index; COPD, chronic obstructive pulmonary disease; CKD, chronic kidney disease; PCI, percutaneous coronary intervention; TG, triglyceride; TC, total cholesterol; Cr, creatinine; UA, uric acid; CK-MB, creatine kinase MB; TnI, troponin I; Mb, myoglobin; BNP, brain natriuretic peptide; LAD, left atrial diameter; LVEF, left ventricular ejection fractions; E/A, ratio early to late diastolic transmitral flow velocity; LVEDD, left ventricular end-diastolic dimension

**Supplementary Fig. 2. Covariate balance plot for assessing balance between POAF duration shorter than 48 hours and POAF longer than 48 hours groups after PSM and IPTW**

After using PSM with IPTW, the SMD for all variables between the POAF duration shorter than 48 hours and POAF longer than 48 hours groups were <0.1.


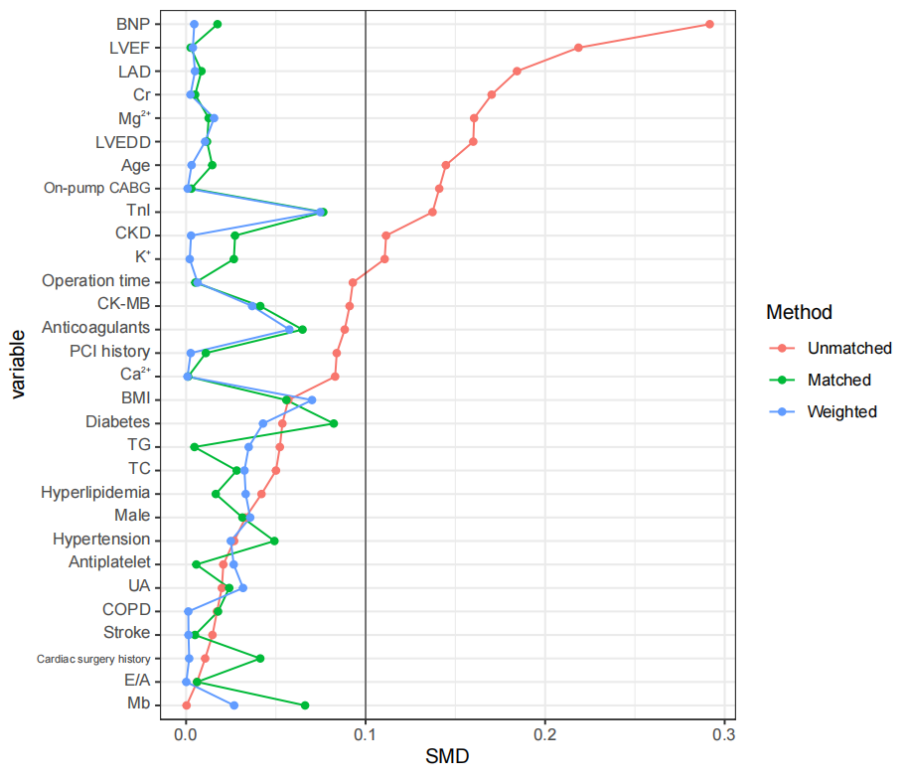


SMD, standardized mean difference; BMI, body mass index; COPD, chronic obstructive pulmonary disease; CKD, chronic kidney disease; PCI, percutaneous coronary intervention; TG, triglyceride; TC, total cholesterol; Cr, creatinine; UA, uric acid; CK-MB, creatine kinase MB; TnI, troponin I; Mb, myoglobin; BNP, brain natriuretic peptide; LAD, left atrial diameter; LVEF, left ventricular ejection fractions; E/A, ratio early to late diastolic transmitral flow velocity; LVEDD, left ventricular end-diastolic dimension
