# Supplementary figures and images for: Association of Postoperative Atrial Fibrillation Duration after Coronary Artery Bypass Grafting with Poor Postoperative Outcomes
Source: Rev Cardiovasc Med. 2024 Mar 8;25(3):98. doi: 10.31083/j.rcm2503098 (PMC11263830; doi:10.31083/j.rcm2503098)

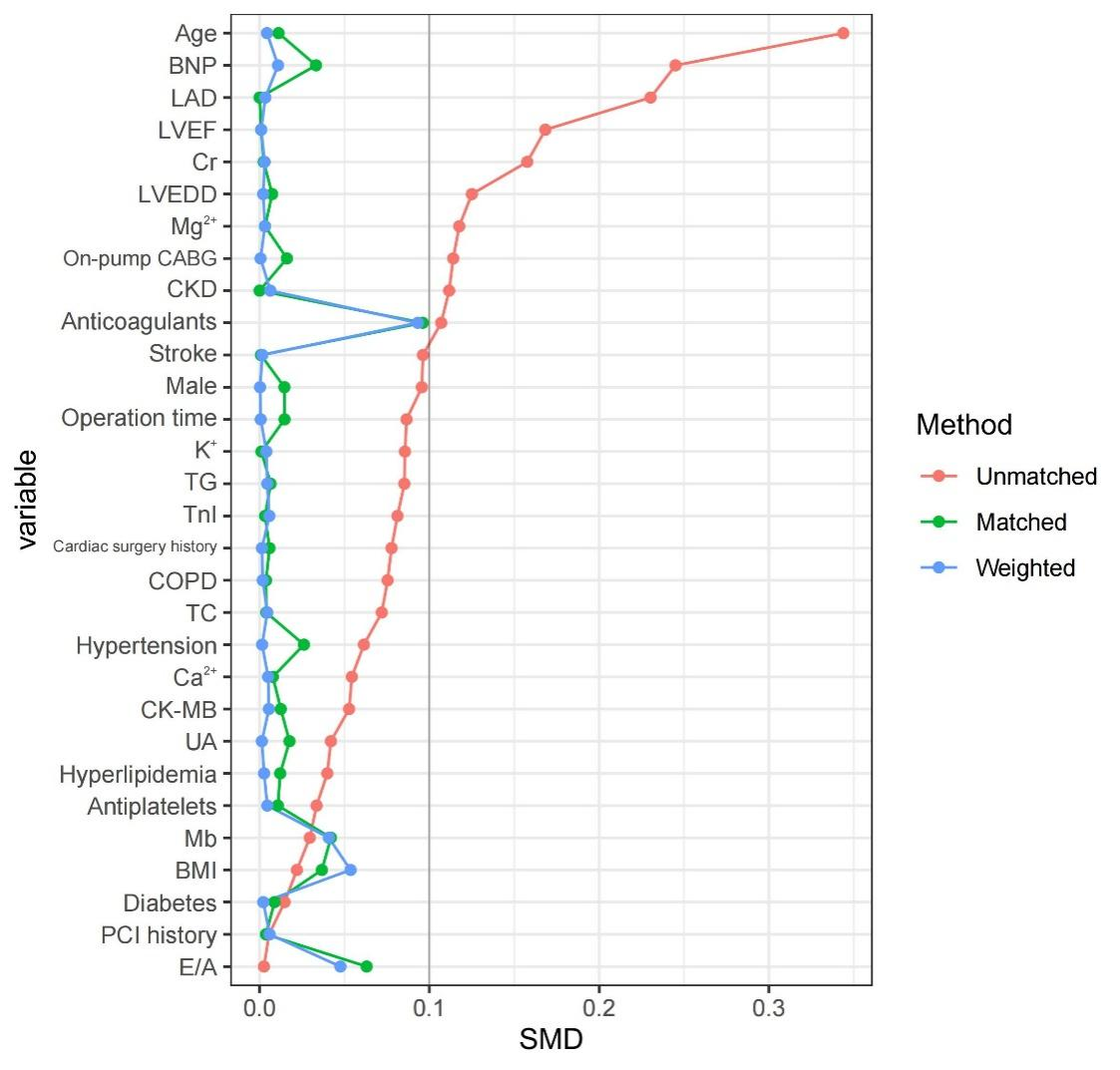

Supplement: Supplementary file 1 [file 2153-8174-25-3-098-s1.zip › Supplementary Fig. 1.tif]

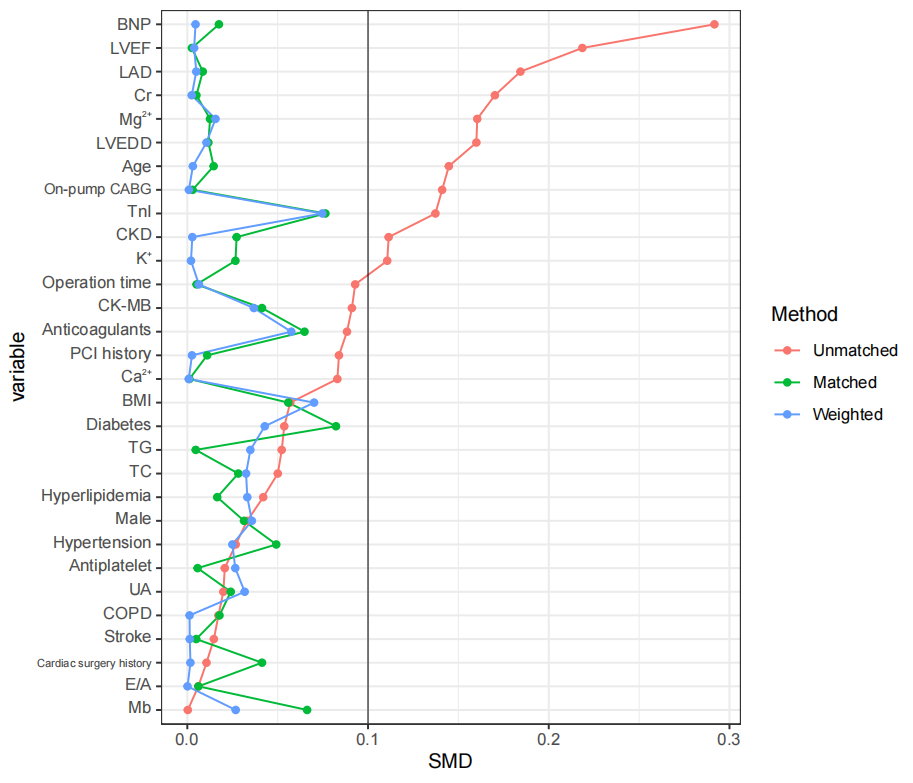

Supplement: Supplementary file 1 [file 2153-8174-25-3-098-s1.zip › Supplementary Fig. 2.tif]
